# Association of Age With SARS-CoV-2 Antibody Response

**DOI:** 10.1001/jamanetworkopen.2021.4302

**Published:** 2021-03-22

**Authors:** He S. Yang, Victoria Costa, Sabrina E. Racine-Brzostek, Karen P. Acker, Jim Yee, Zhengming Chen, Mohsen Karbaschi, Robert Zuk, Sophie Rand, Ashley Sukhu, P. J. Klasse, Melissa M. Cushing, Amy Chadburn, Zhen Zhao

**Affiliations:** 1 Department of Pathology and Laboratory Medicine, Weill Cornell Medicine, New York, New York; 2 NewYork–Presbyterian Hospital/Weill Cornell Medical Campus, New York, New York; 3 Division of Pediatric Infectious Disease, Weill Cornell Medicine, New York, New York; 4 Department of Population Health Sciences, Weill Cornell Medicine, New York, New York; 5 ET HealthCare, Palo Alto, California; 6 Department of Microbiology and Immunology, Weill Cornell Medicine, New York, New York

## Abstract

**Importance:**

Accumulating evidence suggests that children infected with severe acute respiratory syndrome coronavirus 2 (SARS-CoV-2) are more likely to manifest mild symptoms and are at a lower risk of developing severe respiratory disease compared with adults. It remains unknown how the immune response in children differs from that of adolescents and adults.

**Objective:**

To investigate the association of age with the quantity and quality of SARS-CoV-2 antibody responses.

**Design, Setting, and Participants:**

This cross-sectional study used 31 426 SARS-CoV-2 antibody test results from pediatric and adult patients. Data were collected from a New York City hospital from April 9 to August 31, 2020. The semiquantitative immunoglobin (Ig) G levels were compared between 85 pediatric and 3648 adult patients. Further analysis of SARS-CoV-2 antibody profiles was performed on sera from 126 patients aged 1 to 24 years.

**Main Outcomes and Measures:**

SARS-CoV-2 antibody positivity rates and IgG levels were evaluated in patients from a wide range of age groups (1-102 years). SARS-CoV-2 IgG level, total antibody (TAb) level, surrogate neutralizing antibody (SNAb) activity, and antibody binding avidity were compared between children (aged 1-10 years), adolescents (aged 11-18 years), and young adults (aged 19-24 years).

**Results:**

Among 31 426 antibody test results (19 797 [63.0%] female patients), with 1194 pediatric patients (mean [SD] age, 11.0 [5.3] years) and 30 232 adult patients (mean [SD] age, 49.2 [17.1] years), the seroprevalence in the pediatric (197 [16.5%; 95% CI, 14.4%-18.7%]) and adult (5630 [18.6%; 95% CI, 18.2%-19.1%]) patient populations was similar. The SARS-CoV-2 IgG level showed a negative correlation with age in the pediatric population (*r* = −0.45, *P* < .001) and a moderate but positive correlation with age in adults (*r* = 0.24, *P* < .001). Patients aged 19 to 30 years exhibited the lowest IgG levels (eg, aged 25-30 years vs 1-10 years: 99 [44-180] relative fluorescence units [RFU] vs 443 [188-851] RFU). In the subset cohort aged 1 to 24 years, IgG, TAb, SNAb and avidity were negatively correlated with age (eg, IgG: *r* = −0.51; P < .001). Children exhibited higher median (IQR) IgG levels, TAb levels, and SNAb activity compared with adolescents (eg, IgG levels: 473 [233-656] RFU vs 191 [82-349] RFU; *P* < .001) and young adults (eg, IgG levels: 473 [233-656] RFU vs 85 [38-150] RFU; *P* < .001). Adolescents also exhibited higher median (IQR) TAb levels, IgG levels, and SNAb activity than young adults (eg, TAb levels: 961 [290-2074] RFU vs 370 [125-697]; *P* = .006). In addition, children had higher antibody binding avidity compared with young adults, but the difference was not significant.

**Conclusions and Relevance:**

The results of this study suggest that SARS-CoV-2 viral specific antibody response profiles are distinct in different age groups. Age-targeted strategies for disease screening and management as well as vaccine development may be warranted.

## Key Points

**Question:**

Are the quantity and quality of antibodies against severe acute respiratory syndrome coronavirus 2 (SARS-CoV-2) different among children, adolescents, and young adults?

**Findings:**

In this cross-sectional study evaluating 31 426 SARS-CoV-2 antibody tests performed between April 9 and August 31, 2020, immunoglobin G levels were found to vary in different age groups, despite similar seroprevalence in the pediatric and adult patient populations. SARS-CoV-2 immunoglobin G and total antibody levels, neutralizing activity, and avidity exhibited negative correlations with age in patients aged 1 to 24 years.

**Meaning:**

This analysis revealed distinct antibody responses in different age groups, suggesting that age-targeted strategies for disease screening and management as well as vaccine development may be warranted.

## Teaser

This cross-sectional study investigates the association of age with the quantity and quality of severe acute respiratory syndrome coronavirus 2 (SARS-CoV-2) antibody responses.

## Introduction

Coronavirus disease 2019 (COVID-19), caused by severe acute respiratory syndrome coronavirus 2 (SARS-CoV-2), emerged in December 2019 and has caused more than 102 million confirmed cases worldwide as of January 31, 2021.^1^ Despite intensive study throughout the scientific and medical communities, many clinical and biologic aspects of the disease, especially in the pediatric population, have not yet been elucidated. As data emerged from the initial outbreaks in China, the number of COVID-19 cases in children appeared to be low, with reports indicating that less than 1% were patients younger than 10 years, 1.2% were aged between 10 and 19 years, and only 9 patients were infants with mild symptoms.^2^ In the United States, pediatric infection cases comprised only 7% of total cases as of August 2020.^2,3^ The US Centers for Disease Control and Prevention (CDC) reported that, as of September 19, 2020, only 4.1% of the nationally confirmed COVID-19 cases were in school-aged pediatric patients (aged 5-17 years).^4^ Although the causes of these differences remain unclear, most children with SARS-CoV-2 infection are either asymptomatic or exhibit mild symptoms^5,6,7^ and have a low risk of developing severe respiratory disease.^8,9^ The CDC reported that the average weekly incidence of COVID-19 cases among adolescents aged 12 to 17 years was approximately twice that of children aged 5 to 11 years.^4^ Only a relatively small number of pediatric patients have experienced severe disease during the acute phase of COVID-19. However, these patients are at risk of severe complications from multisystem inflammatory syndrome in children (MIS-C), an emerging entity thought to occur as sequelae to acute SARS-CoV-2 infection.^10,11^ Thus, there appears to be differences in pathophysiologic responses to SARS-CoV-2 based on age.

Although the physiologic mechanisms remain unclear, evidence suggests that SARS-CoV-2–specific antibody responses may be different in children and adolescents compared with those in adults,^12^ potentially modulating different clinical manifestations. Controversy exists as to whether children have an attenuated adaptive immune response, leading to tolerance of the SARS-CoV-2 infection,^8^ or if the innate immune response in children plays a more active role against SARS-CoV-2 than in adults.^13^ Additionally, the binding avidity of SARS-CoV-2 viral–specific antibodies, which represents the quality of the antibody response, has not been fully characterized in pediatric patients.

Given that the 2020-2021 school year has resumed, with approximately 56 million school-aged children and adolescents in the United States participating in in-person and/or remote classes, it is imperative to better understand the SARS-CoV-2 viral–specific immune responses in pediatric patients. In this study, the magnitude of total antibody levels, immunoglobin (Ig) G levels, and surrogate neutralizing antibody (SNAb) activities as well as the antibody binding avidity in children, adolescents, and young adults were evaluated. In contrast to other studies, which focused mainly on hospitalized pediatric patients, this study investigated the antibody responses during the convalescent stage of previously asymptomatic or mildly ill nonhospitalized patients, which is more representative of the overall pediatric population of patients with COVID-19.

## Methods

### Patients and Data Sources

This study was performed at NewYork–Presbyterian Hospital/Weill Cornell Medical Center with approval by the institutional review board. A waiver of informed consent was granted by the Weill Cornell Medicine institutional review board because only remnant sera samples were used. This report followed the Strengthening the Reporting of Observational Studies in Epidemiology (STROBE) reporting guideline for cross-sectional studies.

A total of 31 426 SARS-CoV-2 antibody tests were performed between April 9 and August 31, 2020, on serum samples from children (aged 1-18 years) and adults (aged >18 years), including both inpatients and outpatients. Infants younger than 1 year were not included in this study to exclude the possibility of maternal antibody transfer. The results from these tests were used to calculate the SARS-CoV-2 antibody positivity rate for each age group. Among them, positive results of 85 pediatric and 3648 adult cases analyzed using the Pylon COVID-19 IgG assay from April 9 to June 21, 2020, were used to evaluate and compare IgG levels. Results from samples evaluated using the Siemens assay from June 22 to August 31, 2020, were not used for the IgG semiquantitative studies because approximately 50% of the positive results were greater than the upper limit of the reportable range, precluding accurate quantitative antibody comparison. A flowchart of inclusion and exclusion of patient samples is shown in eFigure 1 in the Supplement.

SARS-CoV-2 antibody positive remnant sera were randomly collected in biobank between April 22 and August 31, 2020, from which samples from 126 outpatients aged 1 to 24 years were used for further serology analysis. These patients did not report any COVID-19–like symptoms at the time of antibody testing. The samples were stored at 4 °C for no more than 2 days and then were frozen until further testing. These samples were evaluated for the levels of SARS-CoV-2 IgG, total receptor binding domain (RBD)-binding antibodies (TAb), and SNAb activity as well as the avidity of antibody binding to RBD. The demographic characteristics, medical history, and COVID-19–related clinical and laboratory findings for these 126 patients were obtained from the electronic medical record (EMR; Epic Systems). None of the patients were reported to have MIS-C.

### SARS-CoV-2 Antibody Assays

#### SARS-CoV-2 IgG Assay and SARS-CoV-2 Total Antibody Assay for Clinical Testing

The COVID-19 IgG assay on the Pylon 3D analyzer (ET Healthcare) was used as a laboratory-developed test under New York State Department of Health regulations from April 9 to June 21, 2020. This cyclic-enhanced fluorescence assay targets the S-RBD and recombinant nucleocapsid protein, as described previously.^14^ The ADVIA Centaur SARS-CoV-2 Total antibody assay (COV2T), used from June 22 to Oct 25, 2020, is an US Food and Drug Administration Emergency Use Authorization–approved chemiluminescent immunoassay.^15^ It targets the RBD of the S1 spike protein to detect SARS-CoV-2 antibodies. Both assays were used for clinical COVID-19 serology testing. Method validation was performed to demonstrate equivalence of the 2 assays in reporting positive and negative results (eAppendix in the Supplement).

#### SARS-CoV-2 TAb, SNAb, and Avidity Assays

The TAb and SNAb assays were performed as previously reported.^16,17^ Briefly, the TAb assay measures the overall binding between SARS-CoV-2 antibodies and the RBD of the virus spike (ie, S) protein. The SNAb assay, designed as a competitive binding assay, is based on the SARS-CoV-2 antibody-mediated inhibition of the interaction between the angiotensin-converting enzyme 2 (ACE2) protein and the RBD. The assay readout is the percentage of RBD-ACE2 binding (%B/B0), which is inversely associated with the SNAb activity. The avidity assay measures the dissociation rate of SARS-CoV-2 antibodies from the RBD, which is inversely associated with antibody avidity. The TAb, SNAb, and avidity assays are fully automated on the Pylon 3D analyzer (eAppendix in the Supplement).

### Statistical Analysis

Bivariate associations were evaluated using Fisher exact test between 2 categorical variables while the Kruskal-Wallis test or Wilcoxon rank-sum test were used between numerical variables and categorical variables. The Dunn procedure was used as a posttest for pairwise comparisons of antibody levels between age groups following significant Kruskal-Wallis tests. Correlations between 2 numerical variables were assessed by the Spearman correlation coefficient. Summary statistics are presented as mean with SD or median with interquartile range (IQR) for continuous variables and frequency with proportion for categorical variables. A 2-tailed *P* < .05 was considered statistically significant. Analyses were performed using SAS version 9.4 (SAS Institute) or Prism version 9.0.0 (GraphPad Software).

## Results

### SARS-CoV-2 Antibody Positivity Rates From April 9 to August 31, 2020

**Figure 1.**
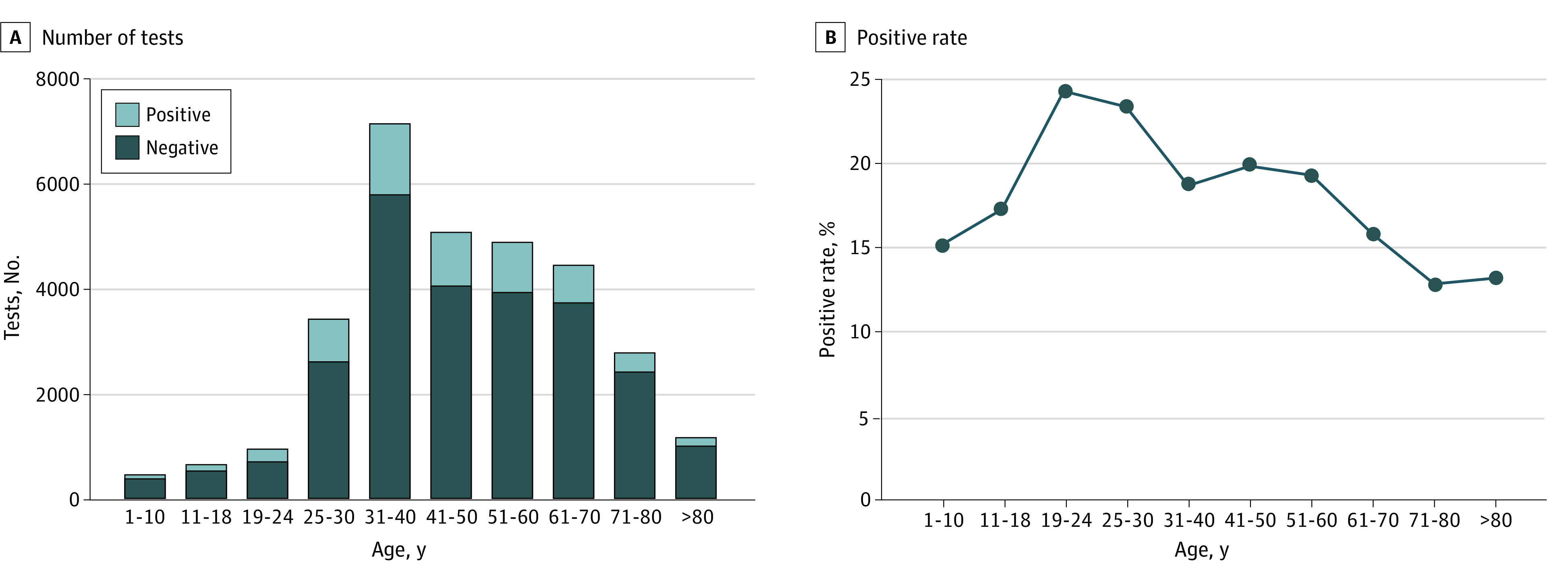
Severe Acute Respiratory Syndrome Coronavirus 2 Antibody Test Frequencies and Positivity Rates from April 9 to August 31, 2020

A total of 31 426 SARS-CoV-2 antibody tests were performed (19 797 [63.0%] female patients), including 1194 tests in pediatric patients (mean [SD] age, 11.0 [5.3] years) and 30 232 in adult patients (mean [SD] age, 49.2 [17.1] years). The testing volume as well as the number of positive results in each age group is shown in Figure 1A. Overall, 197 (16.5%; 95% CI, 14.4%-18.7%) and 5630 (18.6%; 95% CI, 18.2%-19.1%) results were positive in pediatric and adult patients, respectively. The positivity rates for pediatric and adult individuals were not significantly different (*P* = .06). The positivity rates were then evaluated for different age groups (Figure 1B). Young adults, aged 19 to 24 years of age (242 of 990 [24.4%; 95% CI, 21.8%-27.3%]) and those aged 25 to 30 years (816 of 3468 [23.5%, 95% CI, 22.1%-25.0%]) had the highest positivity rates compared with other age groups. Children aged 1 to 10 years (76 of 500 [15.2%; 95% CI, 12.2%-18.6%]), patients aged 61 to 70 years (714 of 4494 [15.9%; 95% CI, 14.8%-17.0%]), patients aged 71 to 80 (365 of 2824 [12.9%; 95% CI, 11.7%-14.2%]), and patients older than 80 years (161 of 1208 [13.3%; 95% CI, 11.5%-15.4%]) had lower positivity rates.

### Comparison of SARS-CoV-2 IgG Levels in Each Age Group

**Figure 2.**
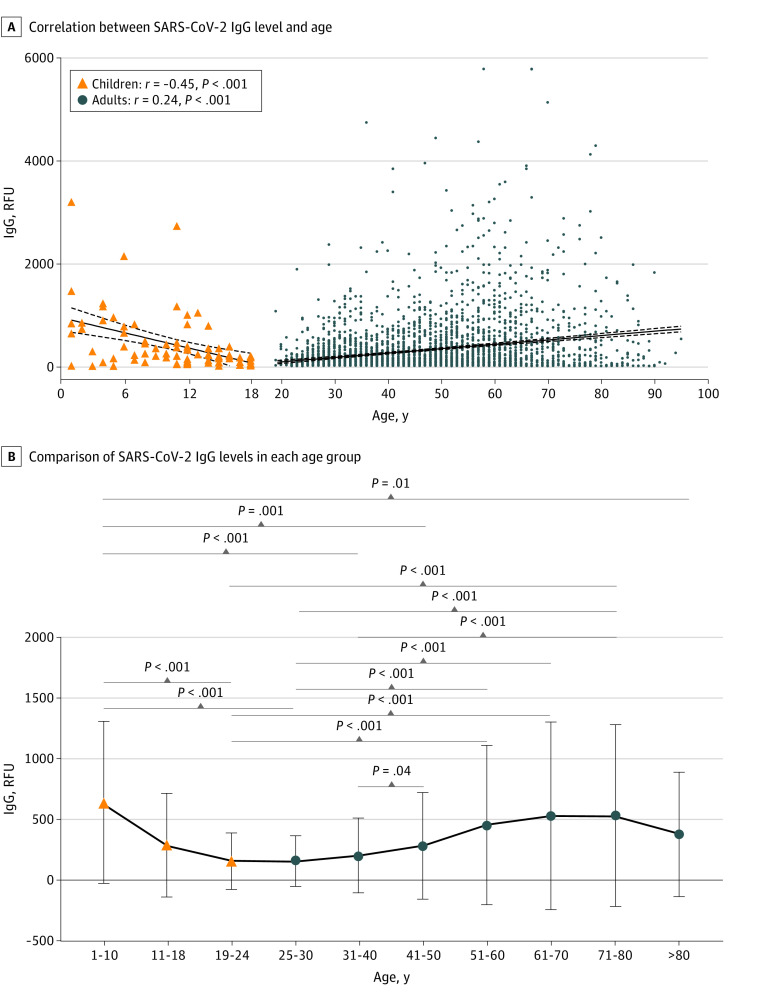
Severe Acute Respiratory Syndrome Coronavirus 2 (SARS-CoV-2) Immunoglobin (Ig) G Levels From 85 Positive Pediatric and 3648 Positive Adult Patient Samples Measured on a Single Platform From April 9 to June 21, 2020 B, Whiskers indicate SD. RFU indicates relative fluorescence units.

We compared the SARS-CoV-2 IgG levels from 85 positive pediatric and 3648 positive adult patient samples measured using a single platform (Pylon 3D), accounting for 43.1% (95% CI, 36.1%-50.4%) and 64.8% (95% CI, 63.5%-66.0%) of the positive pediatric and adult results, respectively. The IgG level in the pediatric population exhibited a moderate but significant negative correlation with age (*r* = −0.45; *P* < .001), and the adult population exhibited a weakly positive correlation with age (*r* = 0.24; *P* < .001) (Figure 2A). Notably, the 32 children aged 1 to 10 years showed significantly higher median (IQR) SARS-CoV-2 IgG levels than the 127 young adults aged 19 to 24 years (443 [188-851] RFU vs 95 [47-180] RFU; *P* < .001), the 611 adults aged 25 to 30 years (99 [44-180] RFU; *P* < .001), the 956 adults aged 31 to 40 year (104 [48-224] RFU; *P* < .001), the 688 adults aged 41 to 50 years (137 [50-319] RFU; *P* = .001), and the 69 patients older than 80 years (165 [24-518] RFU; *P* = .01). Young adults aged 19 to 24 years and 25 to 30 years exhibited the lowest median (IQR) SARS-CoV-2 IgG levels (95 [47-180] RFU and 99 [44-180] RFU, respectively), without any significant difference between these 2 age groups (Figure 2B). Patients aged 19 to 24 years showed significantly lower IgG levels than the 612 adults aged 51 to 60 years (95 [47-180] RFU vs 195 [65-585]; *P* < .001), the 415 aged 61 to 70 years (225 [65-660] RFU; *P* < .001), and the 170 aged 71 to 80 years (233 [62-675] RFU; *P* < .001), and patients aged 25 to 30 years old showed significantly lower median (IQR) IgG levels than adults older than 41 years (eg, vs patients aged 41-50: 99 [44-180] RFU vs 137 [50-319] RFU; *P* < .001) but not those 81 years or older (165 [24-518] RFU; *P* > .99).

### Assessment of SARS-CoV-2 Antibody Quantity and Quality in Children, Adolescents, and Young Adult Patients

**Figure 3.**
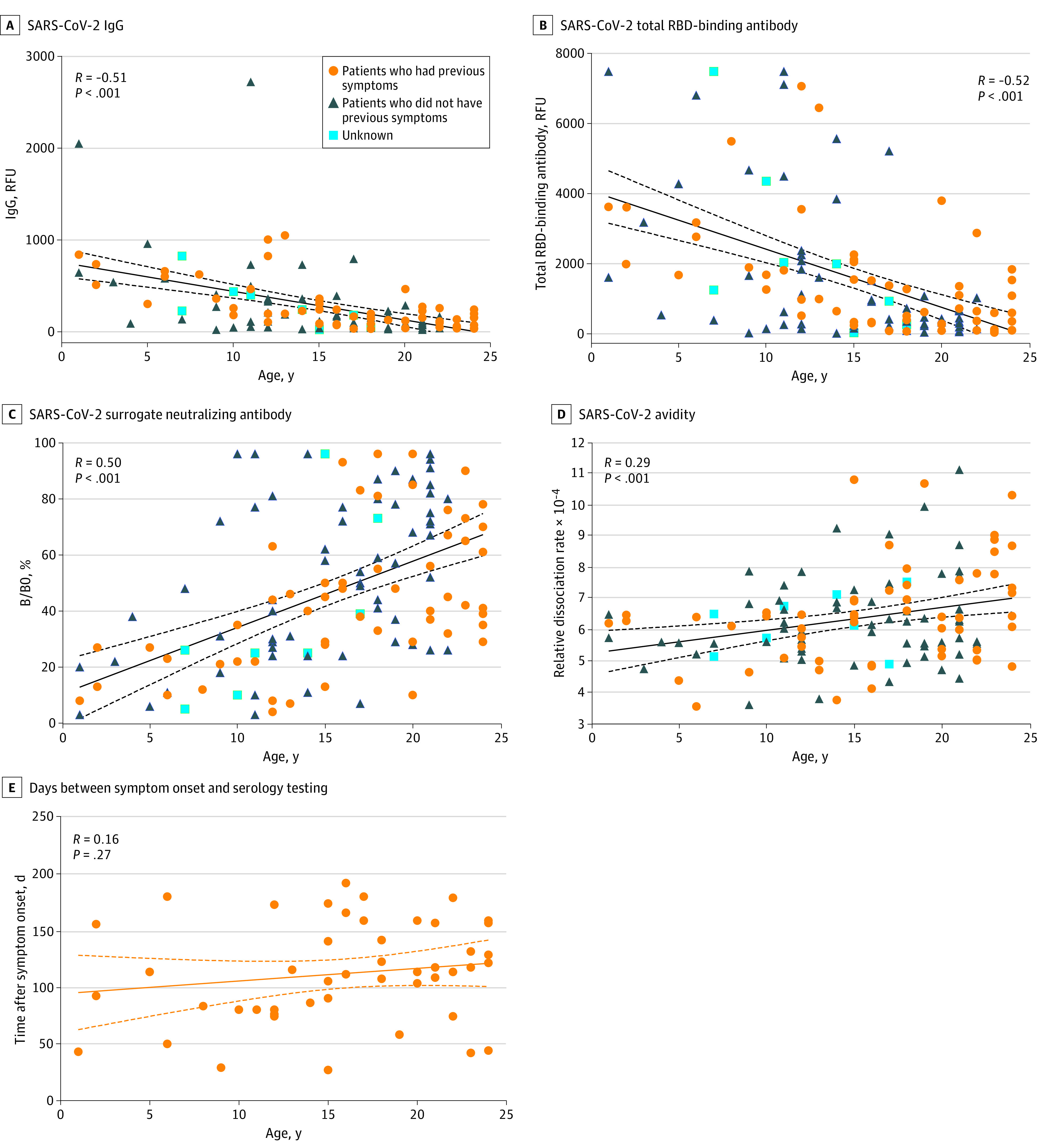
Comprehensive Assessment of Severe Acute Respiratory Syndrome Coronavirus 2 (SARS-CoV-2) Antibodies in 126 Patients Aged 1 to 24 Years %B/B0 indicates percentage of angiotensin-converting enzyme 2 protein and receptor-binding domain (RBD) binding; IgG, immuglobin G; RFU, relative fluorescence units.

We further focused on pediatric patients (aged 1-18 years) and young adults (aged 19-24 years) to understand their characteristic profiles of SARS-CoV-2 antibody responses. More extensive SARS-CoV-2 serologic testing was performed in 126 outpatients aged 1 to 24 years. None of the 126 patients were admitted to the hospital due to COVID-19 prior to serum sample collection, and they were asymptomatic at the time of antibody testing (Table). Of 118 patients with documentation in the EMR, 56 patients (47.5%) were previously symptomatic whereas 62 (52.5%) never had COVID-like symptoms. Nine patients (19.1%) had positive SARS-CoV-2 reverse transcription–polymerase chain reaction testing. Most patients (87 [69.0%]) underwent antibody testing due to previous exposure or previous COVID-19–like symptoms. Other reasons for antibody testing included preprocedural testing (4 [3.2%]), annual checkup (3 [2.4%]), request for attending school or camp (11 [8.7%]), or to confirm prior positive SARS-CoV-2 antibody testing in another hospital (4 [3.2%]). Among patients who had self-reported dates of symptom onset, there was no correlation between age and the days between serology testing and symptom onset (mean (SD) time, 108 [48] days; *r* = 0.16; *P* = .27) (Figure 3E). Characterization of the symptoms and comorbidities in children, adolescents, and young adults are shown in the Table.

**Table.** Demographic and Clinical Characteristics for 126 Patients With Full Antibody Profiles^a^

Characteristic	Patients, No./total No. (%)				*P* value
	Total (N = 126)	Children (n = 24)	Adolescents (n = 58)	Young adults (n = 44)	
Positive RT-PCR test^b^	9/47 (19.1)	1/12 (8.3)	3/29 (10.3)	5/6 (83.3)	<.001
RT-PCR test unavailable	79/126 (62.7)	12/24 (50)	29/58 (50)	38/44 (86.4)	<.001
Race					
Asian	7/124 (5.6)	1/24 (4.2)	4/58 (7.0)	2/43 (4.7)	.04^c^
Black	6/124 (4.8)	1/24 (4.2)	1/58 (1.8)	4/43 (9.3)	
White	55/124 (44.4)	9/24 (37.5)	31/58 (54.4)	15/43 (34.9)	
Declined	36/124 (29.0)	7/24 (29.2)	10/58 (17.5)	19/43 (44.2)	
Other^d^	20/124 (16.1)	6/24 (25.0)	11/58 (19.3)	3/43 (7.0)	
Sex					
Female	64/126 (50.8)	11/24 (45.8)	28/58 (48.3)	25/44 (56.8)	.60
Male	62/126 (49.2)	13/24 (54.2)	30/58 (51.7)	19/44 (43.2)	
Age, y					
Median (range)	16.0 (1.0-24.0)	6.5 (1.0-10.0)	15.0 (11.0- 18.0)	21.0 (19.0-24.0)	NA
Mean (SD)	15.4 (5.9)	6.1 (3.2)	14.6 (2.5)	21.4 (1.6)	NA
Comorbidities					
Type 2 diabetes	2/117 (1.7)	0/21	1/52 (1.9)	1/44 (2.3)	>.99
Hypertension	1/117 (0.9)	0/21	0/52	1/44 (2.3)	.55
Hyperlipidemia	2/117 (1.7)	0/21	0/52	2/44 (4.5)	.31
Allergies	18/117 (15.4)	0/21	10/52 (19.2)	8/44 (18.2)	.08
Asthma	12/117 (10.3)	0/21	5/52 (9.6)	7/44 (15.9)	.14
Chronic lung disease	1/117 (0.9)	0/21	1/52 (1.9)	0/44	>.99
Chronic cardiac disease	5/117 (4.3)	2/21 (9.5)	1/52 (1.9)	2/44 (4.5)	.26
Current immunosuppression	5/117 (4.3)	1/21 (4.8)	1/52 (1.9)	3/44 (6.8)	.49
COVID-19 exposure	65/117 (55.6)	18/21 (85.7)	29/53 (54.7)	18/43 (41.9)	.003
Prior COVID-19 symptoms					
Previously symptomatic	56/118 (47.5)	10/21 (47.6)	23/53 (43.4)	23/44 (52.3)	.69
Fever	25/117 (21.4)	7/21 (33.3)	7/52 (13.5)	11/44 (25)	.13
Dyspnea	2/117 (1.7)	0/21	1/52 (1.9)	1/44 (2.3)	>.99
Cough	22/117 (18.8)	2/21 (9.5)	10/52 (19.2)	10/44 (22.7)	.50
Anosmia or ageusia	14/117 (12)	0/21	7/52 (13.5)	7/44 (15.9)	.16
Sore throat	6/117 (5.1)	0/21	1/52 (1.9)	5/44 (11.4)	.09
Rhinorrhea	10/117 (8.5)	0/21	7/52 (13.5)	3/44 (6.8)	.19
Myalgia	7/117 (6.0)	0/21	1/52 (1.9)	6/44 (13.6)	.05
Fatigue	13/117 (11.1)	0/21	2/52 (3.8)	11/44 (25)	.001
Diarrhea	4/117 (3.4)	0/21	2/52 (3.8)	2/44 (4.5)	>.99
Emesis	2/117 (1.7)	0/21	0/52	2/44 (4.5)	.31
Abdominal pain	3/117 (2.6)	1/21 (4.8)	2/52 (3.8)	0/44	.40
Headache	10/117 (8.5)	0/21	4/52 (7.7)	6/44 (13.6)	.21

Abbreviations: COVID-19, coronavirus disease 2019; NA, not applicable; RT-PCR, reverse transcription–polymerase chain reaction.

^a^ Analyses used nonmissing values in both variables in the 2-way table. *P* values obtained from the statistical tests were by Fisher exact test.

^b^ RT-PCR testing performed prior to serological testing.

^c^ Fisher exact test (excluding other, declined, and unknown groups).

^d^ The other category included American Indian, Hispanic, Native Hawaiian, and others.

**Figure 4.**
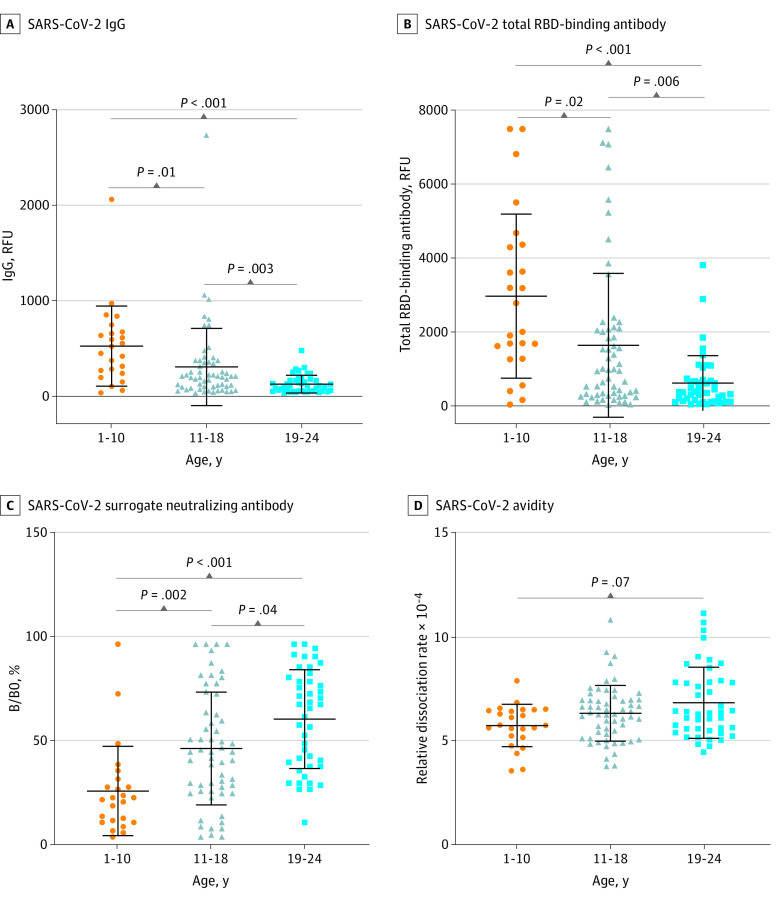
Comparison of Severe Acute Respiratory Syndrome Coronavirus 2 (SARS-CoV-2) Antibodies in Children (Aged 1-10 Years), Adolescents (Aged 11-18 Years), and Young Adults (Aged 19-24 Years) Center horizontal lines indicate mean, with whiskers indicating SD; %B/B0, percentage of angiotensin-converting enzyme 2 protein and receptor-binding domain (RBD) binding; IgG, immunoglobin G; RFU, relative fluorescence units.

Similar to what was seen in the overall patient population (Figure 2), the level of SARS-CoV-2 IgG in this subset patient cohort showed a moderate but significantly negative correlation with age (*r* = −0.51; *P* < .001) (Figure 3A). Children aged 1 to 10 years had significantly higher median (IQR) SARS-CoV-2 IgG levels than adolescents aged 11 to 18 years (473 [233-656] RFU vs. 191 [82-349] RFU; *P* = .01) and young adults aged 19 to 24 years (85 [38-150] RFU; *P* < .001). Adolescents also exhibited a significantly higher IgG level than young adults (191 [82-349] vs 85 [35-150]; *P* = .003) (Figure 4A). Similarly, the SARS-CoV-2 TAb levels in this subset patient cohort showed a negative correlation with age (*r* = −0.52; *P* < .001) (Figure 3B). Pediatric patients, both children aged 1 to 10 years and adolescents aged 11 to 18 years, showed higher median [IQR] SARS-CoV-2 TAb levels than young adults aged 19 to 24 years (children vs young adults: 2393 [1362-4346] RFU vs 370 [125-697] RFU; *P* < .001; adolescents vs young adults: 961 [290-2074] RFU vs 370 [125-697]; *P* = .006).

The SARS-CoV-2 SNAb activity and the binding avidity assays were used to assess the quality of the SARS-CoV-2 antibody. The %B/B0 was positively correlated with age (*r* = 0.50; *P* < .001) (Figure 3C), indicating an inverse correlation between SNAb activity and age. Similar to IgG and TAb, median (IQR) SNAb activities were higher in children (aged 1 to 10 years ) than adolescents (aged 11 to 18 years) (%B/B0: 21.5% [10.3% to 30.0%] vs 44.0% [25.0% to 65.3%]; *P* = .002) and young adults (aged 19 to 24 years) (%B/B0: 66.0% [37.5% to 79.5%]; *P* < .001). Adolescents also exhibited higher median (IQR) SNAb activity than young adults (%B/B0: 44.0% [25.0% to 65.3%] vs 66.0% [37.5% to 79.5%]; *P* = .04) (Figure 4C). The relative dissociation rate between SARS-CoV-2 antibodies and the RBD exhibited a weak but significantly positive correlation with age (*r* = 0.29; *P* < .001) (Figure 3C), indicating a negative correlation between binding avidity and age. While there was no significant difference between each age group, children aged 1 to 10 years old tended to exhibit lower median (IQR) relative dissociation rates and thus higher antibody binding avidity than young adults (5.7 × 10^−4^ [5.2 × 10^−4^ to 6.5 × 10^−4^] vs 6.3 × 10^−4^ [5.5 × 10^−4^ to 7.8 × 10^−4^]; *P* = .07) (Figure 4D).

## Discussion

There is a pressing need to understand the pathophysiologic basis underlying the different disease manifestations of SARS-CoV-2 infection in children compared with adults. However, our understanding of the immune response against SARS-CoV-2 in children and young adults is limited. Although the seroprevalence in the pediatric and adult patient populations was similar, indicating children were as likely as adults to be infected with SARS-CoV-2, we found that SARS-CoV-2 IgG antibody production was distinctly different in children, adolescents, and different age groups of adults. Furthermore, the comprehensive assessment focusing on the SARS-CoV-2 antibody quantitative and qualitative profiles in pediatric patients and young adults revealed key differences in humoral antibody responses against SARS-CoV-2 based on age. Thus, our findings suggest that the differences in the clinical manifestations of COVID-19 in pediatric patients compared with adult patients could be partly due to age-related immune responses.

Similar to what was noted with SARS-CoV and Middle East respiratory syndrome coronavirus infections, SARS-CoV-2 appears to cause fewer symptoms and less severe disease in children than in adults.^9,18^ The exact mechanisms underlying the different SARS-CoV-2 immune responses based on age remain unclear; however, a few possibilities have been suggested. It has been proposed that children may have attenuated immune responses, resulting in tolerance of the virus.^8^ In a 2021 study of 47 pediatric patients (16 with MIS-C and 31 without MIS-C) and 32 adult patients (19 plasma donors and 13 hospitalized patients), Weisberg et al^12^ demonstrated that pediatric patients generated narrower antibody responses to infection in terms of isotypes, which were largely limited to IgG anti-S antibodies, and an overall lower level of neutralizing antibody response than adults. In contrast, other studies have suggested that the milder disease manifestations in children may be due to more active innate immune responses, healthier respiratory tracts due to less exposure to air pollution or cigarette smoke, and fewer comorbidities.^13^ Furthermore, it has also been proposed that trained immunity may play a role^19^ and that innate immune memory generated by other vaccines, in particular by live-attenuated vaccines such as measles, mumps, and rubella, may confer a nonspecific protective effect against SARS-CoV-2.^20^ An additional explanation that has been offered as a possibility for higher antibody levels in children is cross-reactivity with other human coronaviruses, given that multiple putative epitopes for B and T cells are conserved among SARS-CoV-2 and the human coronavirus 0C43 and HKU1.^21^ Here, our results indicate that the anti–SARS-CoV-2 immune response in younger children is more robust than that in adolescents and young adults not only in the magnitude of total antibody and IgG levels but also with respect to functional neutralizing activity. Epidemiologic and clinical data from China, the United States, and other countries suggest that younger children and adolescents may be affected differently by SARS-CoV-2 infection, with children younger than 10 years having milder symptoms than adolescents.^9,13^ This phenomenon coincides with the differences in antibody profile between younger children and adolescents observed in our study, with children showing significantly higher IgG and SNAb than adolescents.

It remains unclear why patients aged 19 to 30 years exhibited lower levels of SARS-CoV-2 IgG antibodies than children and older adults. We would have expected an increase in SARS-CoV-2 antibody levels with increasing age, given that individuals expand their catalog of memory B and T cells through accumulated immunological memory. Furthermore, the inflection of the SARS-CoV-2 antibody response would have been expected to occur at a more advanced age, when the aging immune system fails to mount a robust response to new antigenic challenges. One possibility may lie in the increase in comorbidities, such as obesity, hypertension, or diabetes, commonly associated with increased age in Western society. A multivariate analysis by Racine-Brzostek et al^22^ revealed that when adjusted for age, sex, race, and time from symptom onset to testing, SARS-CoV-2 IgG levels were significantly associated with obesity. It could be postulated that an increased baseline level of proinflammatory cytokines associated with such comorbidities could have a stimulatory effect on the SARS-CoV-2 humoral response.^23^

SARS-CoV-2 antibody avidity in this study showed the same pattern of negative correlation with age as IgG, total, and functional antibody levels. Our analysis demonstrated, for the first time that we know of, a significantly negative correlation between avidity and age in pediatric and young adult cohort. Although not reaching a significant level that may be because of the limited sample size, children exhibited higher antibody binding avidity than young adults. Because antibody avidity in our assay is a measure of the propensity of the antibody to remain bound to RBD,^24^ the higher avidity could augment the antiviral effects of antibodies in children, which could contribute to mitigating clinical manifestations in that group. Measurement of antibody avidity could be of great value to understand the functional efficiency of antibodies and provide insights into antibody-mediated immune protection. It may also be of use in the selection of plasma donors for the treatment of COVID-19 and will be important in understanding differences in responses elicited by the different SARS-CoV-2 vaccine.

### Limitations

Our study has limitations. First, there may be selection bias in terms of who is being tested for SARS-CoV-2 antibodies. As the study was retrospective and reliant on existing data, it was reliant on the ordering clinician for justification of testing for SARS-CoV-2 antibodies. Children and adults were tested for various reasons, as previously mentioned. Second, the time between disease onset and antibody testing was unknown for most patients, especially in asymptomatic patients. However, as serum samples were collected between April and August 2020 (a few months after the initial outbreak of COVID-19 in New York City), the patients who had positive SARS-CoV-2 serology were in their early recovery phase. Furthermore, longitudinal samples were not available for our patients and thus dynamic changes in the magnitude and avidity of SARS-CoV-2 antibodies could not be investigated. Fourth, young children may not be able to describe their symptoms as clearly as adolescents or adults, and the study was reliant on parental reporting.

## Conclusions

The findings of this study suggest that there are distinct SARS-CoV-2 viral–specific antibody response profiles that vary based on age, with younger children exhibiting higher levels of IgG, total, and functional antibody activity than adolescents and young adults. Our data could partly explain the overall lower rate of symptoms and cases of severe disease in children infected with SARS-CoV-2. However, the lower incidence of symptoms and decreased disease severity in pediatric patients raises the possibility that this population could represent an important reservoir for viral transmission in the community. Thus, increased screening of school-aged children, even those without overt symptoms or exposure, may be an important step in curbing the pandemic. Furthermore, these differences in disease manifestations based on age suggest the need for age-targeted strategies for treatment. Measurements of antibody quantity and quality, as described here, could also assist in guiding rational vaccine choice and deployment based on age.

## Notes

Supplement. **eAppendix.** Supplementary Methods and Results **eFigure 1. **Inclusion and Exclusion Flowchart of Patient Samples in the Study
