# Supplementary material for: Association of Age With SARS-CoV-2 Antibody Response
Source: JAMA Netw Open. 2021 Mar 22;4(3):e214302. doi: 10.1001/jamanetworkopen.2021.4302 (PMC7985726; doi:10.1001/jamanetworkopen.2021.4302)
Supplement: Supplement. — eAppendix. Supplementary Methods and Results eFigure 1. Inclusion and Exclusion Flowchart of Patient Samples in the Study [file jamanetwopen-e214302-s001.pdf]

Supplemental Online Content

Yang HS, Costa V, Racine-Brzostek SE, et al. Association of age with SARS-CoV-2 antibody response. *JAMA Netw Open*. 2021;4(3):e214302. doi:10.1001/jamanetworkopen.2021.4302

eAppendix. Supplementary Methods and Results

eFigure 1. Inclusion and Exclusion Flowchart of Patient Samples in the Study

This supplemental material has been provided by the authors to give readers additional information about their work.

© 2021 Yang HS et al. *JAMA Network Open.*

eAppendix. Supplementary Methods

SARS-CoV-2 IgG Cyclic Enhancement Fluorescence Assay (CEFA)

The Pylon COVID-10 IgG assay measures the IgG antibodies against SARS-CoV-2 in serum on the Pylon 3D analyzer ((ET Healthcare, Palo Alto, CA). The antigen for the CEFA method is the S-Receptor Binding Domain (RBD) and recombinant Nucleocapsid Protein (NP). The index value (IV) was determined by the instrument readout of the test sample divided by instrument readout at cut off. Samples with an IV ≥ 1 were designated as positive. 20-day precision study revealed 5.6% for the positive QC (IV = 134) and 19.8% for the negative QC (IV = 0.7). Dynamic range of this assay is 7-5788 total fluorescent unit (r^2 = 0.99).

SARS-CoV-2 total receptor-binding domain (RBD)-binding antibody (TAb)

The TAb assay measures the overall binding between SARS-CoV-2 antibodies and the RBD of the virus spike (S) protein. The assay uses RBD pre-coated probes and preloaded reagent strips. TAb assay was performed as previously reported[18]. The TAb assay is fully automated on the Pylon 3D analyzer (ET Healthcare, Palo Alto, CA). Dynamic range of the TAb assay is 8-7494 RFU (r^2 = 0.98). The limit of detection of the TAb assay is 5 RFU, and the cutoff is 20 RFU. Imprecision of the assay was determined by running the high and low level of pooled patient samples for 5 times per day for 5 different days, and CV was 6.9% and 5.3%, respectively.

SARS-CoV-2 surrogate neutralizing antibody assay (SNAb)

Designed as a competitive binding assay, the SNAb is based on the SARS-CoV-2 antibody-mediated inhibition of the interaction between the angiotension-converting enzyme 2 (ACE2) protein and the RBD. The SNAb assay is also fully automated on the Pylon 3D analyzer (ET Healthcare, Palo Alto, CA). The assay readout is the percentage of RBD-ACE2 binding, which inversely correlates with the SNAb binding inhibition (surrogate neutralizing antibody activity); the assay is performed as previously described[18]. Dynamic range of the SNAb assay is 3-96% %B/B0 ((r^2 = 0.99). The limit of detection of the SNAb assay is 96%. Cutoff is 85% %B/B0. Impression of the SNAb assay was determined by running the high and low level of the pooled patient samples for 5 times per day for 5 days, and CV was 7.7% and 11.5%, respectively.

SARS-CoV-2 antibody avidity assay

The avidity assay measures the rate of SARS-CoV-2 specific antibodies dissociated from RBD, which is inversely correlated with the antibody avidity. In this assay, an RBD pre-coated probe is sequentially incubated in diluted serum sample, biotinylated RBD and Streptavidin-Cy5 conjugate to detect SARS-CoV-2 antibodies. After each incubation step, the probe is washed in wash buffer. The fluorescent signal before dissociation is the measured (*Signal_0*), after which the probe with the immobilized immunocomplex goes into multiple repetitive dissociation cycles of incubation in PBST dissociation buffer (PBS with 0.05% Tween20, pH 7.4). The fluorescent signal is measured at the end of each interval ($Signal_t$).

Dissociation of Covid-19 antibody from either the coated RBD on the probe surface or the biotinylated RBD (linked to Cy5-Streptavidin) could result in the separation of Cy5 from the probe surface and thus,

© 2021 Yang HS et al. *JAMA Network Open.*

loss of the signal. Fluorescent signal directly correlates to the amount of antibody bound on the probe at the time of measurement. Therefore, the amount of antibody bound on the probe surface through dissociation relative to the total amount of antibody on the probe before dissociation starts ([bound]/[total]) is expressed by the ratio of fluorescent signal at each time point through dissociation over the fluorescent signal before dissociation starts (*Signal_t* / *Signal_0*). The dissociation profile is constructed by plotting the fluorescent signal ratio as a function of time. The relative dissociation rate (dR) is calculated by fitting the first order rate equation to the dissociation profile: $\mathrm{Ln}(Signal\_t / Signal\_0) = \mathrm{Ln}([bound]/[total]) = -dR\ t$.

Siemens SARS-CoV-2 Total (COV2T) assay

The COV2T assay is a chemiluminescent immunoassay intended for qualitative detection of total antibodies (including IgG and IgM) to SARS-CoV-2 in human serum and plasma on the Siemens ADVIA Centaur instrument. The dynamic range of the COV2T assay is 0-10 index value (IV). Samples with an IV ≥ 1 were designated as positive. Positive (IV = 2.4) and negative quality control (IV = 0.07) materials were run 5 times per day for 5 days, and CV was 1.5% and 33.4%, respectively. Method comparison was performed and demonstrated equivalence of the two assays in reporting positive and negative. Comparison of 40 positive and 60 negative samples between the Siemens COV2T assay and the Pylon IgG assay revealed a positive agreement rate of 97.5% (the discrepant sample was only positive for IgM) and a negative agreement rate of 100%.

Supplemental results:

Based on the chart review, of 126 patients in the subset patient cohort, 56 patients were previously symptomatic whereas 62 patients never had COVID-like symptoms. Patients who previously had symptoms exhibited significantly higher levels of TAb, IgG, and SNAb activities than those who never had symptoms ($p < 0.001$ for each comparison). However, avidity was not different between patients with or without previous symptoms ($p = 0.15$).

© 2021 Yang HS et al. *JAMA Network Open.*

eFigure 1. Inclusion and Exclusion Flowchart of Patient Samples in the Study

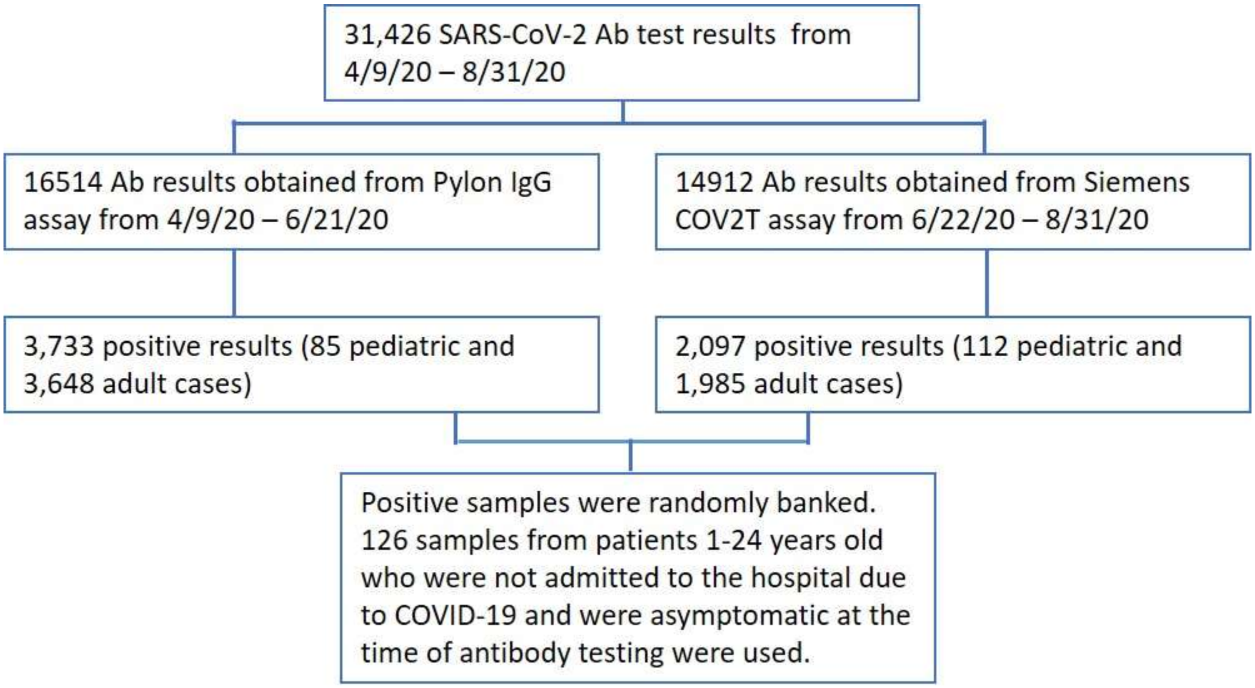

© 2021 Yang HS et al. *JAMA Network Open.*
